# Neuroscience of Meditation

**DOI:** 10.1100/tsw.2006.353

**Published:** 2006-11-16

**Authors:** Vinod D. Deshmukh

**Affiliations:** Flagler Hospital, 300 Health Park Boulevard, Suite 5010, St Augustine, FL 32086, USA

**Keywords:** brain-mind-reality, self-consciousness, reality-consciousness, attention, presence, STEP model of self, simultaneous and sequential consciousness, non-REM alertness, meditation, self, reality, consciousness, neuroscience, inter-attentional awareness

## Abstract

*Dhyana-Yoga* is a Sanskrit word for the ancient discipline of meditation, as a means to *Samadhi* or enlightenment. Samadhi is a self-absorptive, adaptive state with realization of ones being in harmony with reality. It is unitive, undifferentiated, reality-consciousness, an essential being, which can only be experienced by spontaneous intuition and self-understanding. Modern neuroscience can help us to better understand Dhyana-Yoga. This article discusses topics including brain-mind-reality, consciousness, attention, emotional intelligence, sense of self, meditative mind, and meditative brain. A new hypothesis is proposed for a better understanding of the meditative mind. Meditation is an art of being serene and alert in the present moment, instead of constantly struggling to change or to become. It is an art of efficient management of attentional energy with total engagement (*poornata*, presence, mindfulness) or disengagement (*shunyata*, silence, emptiness). In both states, there is an experience of spontaneous unity with no sense of situational interactive self or personal time. It is a simultaneous, participatory consciousness rather than a dualistic, sequential attentiveness. There is a natural sense of well being with self-understanding, spontaneous joy, serenity, freedom, and self-fulfillment. It is where the ultimate pursuit of happiness and the search for meaning of life resolve. One realizes the truth of ones harmonious being in nature and nature in oneself. It is being alive at its fullest, when each conscious moment becomes a dynamic process of discovery and continuous learning of the ever-new unfolding reality.

## INTRODUCTION

*Dhyana-Yoga* is a *Sanskrit* word for meditation derived from the verb *Dhyayati*, which means to meditate, ponder, or self-reflect. By practice of *dhyana*, one can achieve a state of intuitive, self-absorptive consciousness with serenity and presence of mind, which may lead to *samadhi* or enlightenment. Such a mental state is conducive to self-integration and understanding of the truth of one's being in reality and reality in oneself. The word *Dhyana* was transformed into *Jhana* in Pali, *Ch'an* in Chinese, and *Zenna* or *Zen* in Japanese language. The word *Yoga* is derived from the verb *Yuj*, which means to integrate or unite. *Yoga* is a way of integration through self-realization. It is a system of discipline that facilitates the ultimate goal of an adaptive, creative, and enlightened living. Neuroscience has advanced considerably in understanding the mental phenomena of attention, memory, cognition, consciousness, the sense of self, and social-emotional intelligence. These different domains of information processing can be understood in terms of activation or inhibition of specific brain structures and their connections.

## BRAIN-MIND-REALITY

The brain is an interface that mediates interactions between the internal milieu of the organism and its environment. In a broad sense, mental activity goes on within the organism's brain, with episodic interactions with what is happening outside. Most of the current neuroscientists disagree with a view that mind is an entity, independent of the human brain. The current neurobiological perspective suggests a unified view of the brain-mind-reality. One of the strong proponents of such a neurobiological view was Young[1]. He stated: "The point for us is that all intellectual and emotional powers require activity in the brain and we are utterly dependent on it. No brain – no mind, no intellect, no nothing. The clinical evidence does not support the idea that there is some entity or spirit that can exist apart from the brain". In reference to "sublime feeling", Churchland[2] made a valid point: "Real, the feelings certainly are. Worthy, in and of themselves, they also are – the more so if they inspire kindness and virtue, the less so if they inspire cruelty and terror. Do we trivialize a sublime feeling if we appreciate its dependence on the brain? Not in the least. Its significance does not depend on its being a soul state rather than a brain state. Indeed, self-deception is no virtue, and upon reflection, we may find it unworthy to give our experiences, wonderful though they may be, an inflated cosmic significance." Damasio[3] suggested a two-part solution to the puzzle of brain-mind-reality: "The first is the problem of how we generate what I call a 'movie-in-the-brain', an integrated composite of images in diverse sensory modalities – visual, auditory, tactile, olfactory, and so on. The second is the problem of 'self', which pertains to how we generate automatically the sense that we own the 'movie-in-the-brain'." His hypothesis is: "The brain uses mapping structures informed by both maps of the organism and maps of objects, to create a fresh, second-order representation, which indicates that the organism as represented in the brain is involved in interacting with the object. The second-order representation occurs in neural structures such as thalamus and cingulate cortices."

Cariani[4] emphasized the regenerative aspect of this biophysical process. He drew a parallel between life and mind, stating: "Living organization entails continual regeneration of material parts and functional relations (self-production). Similarly, dynamic stability of informational states in brains may entail coherent self-regenerating patterns of neural signals. If mind is the functional organization of the nervous system, then mental states can be seen as switching between alternate sets of stable, self-regenerative neural signal productions."

The neuroecological perspective, considering organism and environment as a single, functional system was emphasized by Jarvilehto[5]: "In any functional sense, organism and environment are inseparable and form only one unitary system. The organism cannot exist without the environment, and the environment has descriptive properties only if connected to the organism." Goerner and Combs[6] summarized: "The evolution of consciousness is seen in the context of energy-driven evolution, where energy and information are understood as two sides of the same coin. Arthem and Liljenstrom[7] argued that "Adaptation and learning are widespread and old properties of living organisms, even as old as life itself. Cognition or knowledge processing mediated by a centralized nervous system… The evolutionary perspective suggests that consciousness can be understood as a biological phenomenon."

Recently, Ventegodt et al.[8] published a holistic perspective of biology and medicine. In a series of four articles on human development, they proposed the application of an energetic-informational interpretation of reality or a holistic perspective to many natural sciences[9,10,11,12]. In discussing the structure and function of the human brain, they suggest: "The concept of information-directed self-organization gives a special meaning to the function of the brain. The brain is basically connected to itself, to the outer world through the senses, to the body through the nerves, and to the wholeness through the coherence given from the fundamental structure of the world."

The following are some of the most interesting and well-established facts in neuroscience, which shed light on the brain and mind relationship:

- A well-functioning brain is essential for human mental activity and behavior. Mind and consciousness are functions of a living brain. They cease to exist when the brain fails to function. There is no bio-psycho-physical afterlife. An individual survives only through one’s progeny and personal legacy.
- Human sleep, dream, and wakefulness are dependent on a well-functioning centrencephalic, reticular activating system. This system consists of chemically specific clusters of neurons that generate specific neurotransmitters like acetyl choline, norepinephrine, dopamine, serotonin, histamine, glutamate, GABA, and orexin. These neurotransmitters activate or inhibit the cerebral cortex and spinal cord. The sleep-wake cycle is regulated by a circadian clock in the suprachiasmatic nucleus of the hypothalamus. It is reset daily at dawn and dusk by the environmental and internal stimuli or milieu.
- The brainstem regulates most of the vital functions of the body including heart rhythm, circulation, breathing, swallowing, eye and head movements, phonation, and automatic movements like walking, running, etc.
- The cerebral cortex is essential for higher mental functions. It consists of two cerebral hemispheres with specialized areas devoted to specific functions. In general, the left hemisphere, in a right-handed person, specializes in language processing, whereas the right hemisphere specializes in visuospacial and auditory processing, attention, and inhibition of behaviors.
- The frontal lobes at the front of the brain modulate voluntary movement; speech; working memory; planning of complex, sequential actions; emotions; and self-reflection and personality traits. The temporal lobes**,** along with the limbic system (hypothalamus, amygdala, hippocampus, cingulate gyrus, orbito-frontal cortex) are involved in emotion, motivation, and memory processing. The parietal-temporal-occipital lobes at the back part of the brain have specialized areas dedicated to the integration of perceptions like vision, audition and touch, speech comprehension, object and face recognition, space perception, and processing of colors, numbers, mathematics, and music. These cortical areas have two streams of information processing: a ventral pathway for vision, the “what” processing, which is memory dependent, slow, and sequential; and a dorsal pathway for action in real time, the “where” processing, which is perception dependent, quick, and simultaneous[13,14].

## CONSCIOUSNESS

All of us know, from our experience, what it is like to be conscious. This is our subjective, first-person perspective. We can also observe a person and his behavior, and tell whether he is conscious or not; this is an objective, third-person perspective. When we direct our attention to objects around us, the knowing process creates a sense of duality of the attended and the attendee, or the knower and the known.

In neuroscience, there have been many attempts to define consciousness. Mahowald[15]: “Consciousness is a primary function and activity of the human brain itself. Mandatory for a neural basis of consciousness is the existence of a constantly present, spontaneously generated oscillatory neural activity (rhythmic impulse or *spandanam*). Consciousness is simply the brain’s integration and interpretation of all information, available to it, at any given point in time. During wakefulness, the sensory information is due to environmental stimulation. During sleep, consciousness is attempting to “make sense” of the internally generated brain activity.” Tononi and Edelman[16] stated: “conscious experience is integrated (each conscious scene is unified) and at the same time it is highly differentiated (within a short time, one can experience any of a huge number of different conscious states). Integration is a property shared by every conscious experience irrespective of its specific content: Each conscious state comprises a single ‘scene’. The key neural mechanism underlying conscious experience is the reentrant, interaction between posterior thalamocortical areas involved in perceptual categorization and anterior areas related to memory, value and planning of action. Such interactions among neural groups may be necessary to generate a unified neural process, corresponding to a multimodal conscious scene… (Such) a unified neural process of high complexity is termed the 'dynamic core'."

Jones[17] reviewed the neural basis of consciousness across the sleep-wake cycle. In summary, consciousness depends on a global, coherent activation of thalamocortical systems by the brainstem reticular activating system during waking. Posner and Rothbart[18] stated: "Consciousness includes awareness of the world, feelings of control over one's behavior and mentation (volition), and the notion of a continuing self. Focal, executive attention is used to control details of awareness and is closely related to volition. The executive attention network is associated with voluntary ability to select among competing items, to correct error and to regulate emotions. Recent neuroimaging studies suggest that these functions involve areas of the anterior cingulate… Executive attention appears to develop first to regulate distress during the first year of life. During later childhood the ability to regulate conflict among competing stimuli builds upon the earlier cingulate anatomy to provide a means of cognitive control. (Thus,) the executive attention networks serve to regulate both cognition and emotion."

Zeman[19] defines "consciousness as i) the waking state; ii) the experience; and iii) the possession of any mental state… Self-consciousness refers to i) the ability to detect our own sensations and recall our recent (experiences and) actions; ii) self-recognition; iii) the awareness of awareness (meta-awareness)*;* and iv) self-knowledge in the broadest sense." Parvizi and Damasio[20] emphasized the role of brainstem reticular formation and other nuclei as the somatosensing structures necessary for core consciousness and core self to emerge. They describe: "The basic, core consciousness, is placed in the context of life regulation; it is seen as yet another level of biological processing aimed at ensuring the homeostatic balance of the living organism …Core consciousness is conceived as the imaged relationship of the interaction between an object and the changed organism state (that) it causes."

Bleck[21] defined "consciousness as a set of neural processes that allow an individual to perceive, comprehend, and act on the internal and external environments." Consciousness has three components. (a) Arousal: It describes the degree to which the individual appears to be able to interact with these environments… It requires the interplay of both the (midbrain) reticular formation and the cerebral hemispheres. The midbrain may be viewed as a driving center for the higher structures. (b) Awareness: It reflects the depth and content of the aroused state. It is dependent on arousal, because one who cannot be aroused appears to lack awareness. It does not imply any specificity for the modality of stimulation. This stimulation may be external (e.g., auditory) or internal (e.g., thirst)… Awareness implies that the individual is not only alert but is cognizant of self and surroundings. Interaction of the cerebral cortex and the reticular system is required for the individual to be aware. (c) Attention: It depends on awareness and implies the ability to respond to particular types of stimuli… Attention to specific aspects of the perceived universe depends on both awareness as a general property and on the specific anatomical structures that mediate the sensory phenomena involved. Usually, the right parietal lobe function is crucial for attention to both hemi-perceptual spaces and its lesion can cause a left hemi-perceptual neglect syndrome. Evans[22] suggested that "there may be two separate thalamocortical networks; one relating to cortical and thalamic areas with specific functions and the other global, involving all cortical areas and so-called 'non-specific' thalamic nuclei. The global system might function as a cortical integrating mechanism permitting the spread of information between the specific cortical areas and thus underlying awareness. The global system may also be responsible for much of the spontaneous and evoked electrical activity of the brain."

Crick et al.[23] stated that the neuronal basis of consciousness is the greatest challenge to the scientific world-view. They further commented that "the function of consciousness is to provide a general-purpose mode of perception that can cope with complicated situations and select one of many possible reactions, including silent thought. There would be a considerable evolutionary advantage to a creature having these two interacting modes: one to provide a limited number of rapid, stereotyped unconscious responses and the other, parallel but slower, for more measured conscious responses to more complex situations… One can be clearly conscious of what one is attending to, and one can easily remember recently attended items, but consciousness for the non-attended parts of the scene is only vague, if present at all… Thus, seeing involves unconscious competition between various tentative interpretations, a winner-take-all process involving many cortical areas; the neural activity associated with this winning coalition would be sufficient to mediate the specific content of consciousness at that time. After an interval, another winning coalition emerges, based in part on the ongoing input."

Ramachandran[24] summarized his views: "There are really two problems here - the problem of the subjective sensations or qualia and the problem of the self. The qualia question is how does the flux of ions in little bits of jelly – the neurons – in our brains give rise to the redness of red, the flavor of Marmite or paneer tikka masala or wine? Matter and mind seem so utterly unlike each other. One way out of this dilemma is to think of them really as two different ways of describing the world, each of which is complete in itself. Just as we can describe light as made up either of particles or as waves – and there is no point in asking which description is correct, because they both are, even though the two seem utterly dissimilar – the same may be true of mental and physical events in the brain."

Dehaene and Changeux[25] postulated that "a sensory stimulus becomes (consciously) reportable when its neural representation in posterior areas is amplified and made available, via long-distance neural connections, to multiple distant sites. Neuronal simulations of this conscious neuronal workspace model suggest that a dynamical phase transition, corresponding to a sudden, all-or-none "ignition" of a large-scale reverberating and self-sustained assembly, underlies the threshold of consciousness. Below this threshold, fast propagation of sensory information remains possible but without global ignition." The conscious global neuronal workspace includes neurons from prefrontal, cingulate, and parietal cortex. "Two modes of conscious access to sensory information should be distinguished: a top-down mode, in which work space neurons become spontaneously activated and selectively amplify a sensory signal, and a bottom-up mode, in which initially unattended sensory signals carry sufficient strength to cause a reorienting of top-down amplification toward them."

Cantero and Atienza[26] reviewed the role of neural synchronization in the emergence of cognition across the wake-sleep cycle. In discussing large-scale cortical integration, they quote supportive evidence, for instance, face perception induced a long-distance pattern of synchronization between parieto-occipital and fronto-temporal regions within the gamma range (30–40 Hz) corresponding to the moment of perception itself. Such synchronization was absent during the no-perception condition.

Kaufmann et al.[27] studied the transition from wakefulness to various stages of NREM sleep, using the functional MRI and EEG. "During all stages of NREM sleep, reduced activity was seen in midbrain, hypothalamus, thalamus, caudate nucleus and limbic lobe. Sleep stage 2, which is linked to a loss of self-awareness, is associated with signal decreases in cingulate cortex, right insula and adjacent temporal lobe, the inferior parietal lobule and the inferior and middle frontal gyri... The hypothalamus is of central importance for sleep regulation, involved in a reciprocal network of wake-promoting nuclei in the brainstem and hypothalamus and sleep-promoting neurons inside the hypothalamic ventrolateral preoptic nucleus… In the default mode of brain function during the resting state, it is assumed that the cingulate cortex subserve a network with greater activity during resting state than during cognitive tasks. The activation of these areas is explained by alertness in expectation of environmental stimuli that are only reduced when focusing attention occurs in the context of a specific task. (This is similar to the attenuation of EEG alpha rhythm from a mentally resting and eye-closed condition to a mentally engaged and eye-open condition)."

Balkin et al.[28] studied the process of awakening from stage 2 sleep, measuring regional cerebral blood flow, using a PET scanning method. They found that awakening from sleep entails rapid re-establishment of consciousness, followed by relatively slow (20–30 min later) re-establishment of alertness... Cerebral blood flow (CBF) was most rapidly established in centrencephalic regions (e.g., brainstem and thalamus), and across ensuing 15 min of wakefulness, further increases in CBF were evident primarily in the anterior cortical regions (mediating alertness).

## ATTENTION

Human attention depends on three neural networks[29,30]: (a) a subcortical, alerting network that maintains an adequate level of conscious arousal for cognitive processing; (b) a posterior cortical orienting network, which directs the body, head, ears, and eyes towards an interesting/alarming object, activity, or experience, chosen to be attended to; and (c) an anterior cortical selective-executive network, which enhances the perception of the selected target and suppresses interference by distracters like other objects, events, images, and thoughts. Attention can be focused on a specific place, time, and modality like vision, audition, thought, etc. Focusing in space enhances the processing of that region of interest, whereas, focusing in time enhances the processing of events at the present moment. It is the basis of presence or present mindedness[70], which is also known as mindfulness in Zen literature. Attention functions in two different modes. The mnemonic mode is dominated by memories, associations, and ruminative thinking. The presence mode is the key to adaptive mental excellence. It requires total attention to the present reality with freedom from the past memories, biases, preferences, and projections[31]. When we direct our attention to objects around us, the knowing process creates a sense of duality of the knower and known.

During meditation, when situational conflicts, desires, and needs are resolved, and when there is no need to attend to anything specific, one can return to a naturally restful state of undistracted, nonspecific (*nirguna*) awareness, with no sense of ego or authorship. Such spontaneous, self-absorptive, nondual state of being is known as *Turiya*[32]. It is the primal ground of all of our conscious experience. It is nonverbal, ineffable, and indefinable, but it is verifiable intuitively by personal experience.

Manly et al.[33] described the characteristics of an absent mind. “To be absent-minded is to be inattentive to the ongoing activity, to lose track of current aims and to become distracted from intended thought or action by salient but (currently) irrelevant stimuli.” Assmus et al.[34] make an important point that perception of space and time is integrated in the left inferior parietal cortex or the supramarginal gyrus. Robertson and Garavan[35] defined vigilant attention as a continuous inhibition of task irrelevances... (This is similar to the classical definition of *Yoga* by *Patanjali*: *Yogah chitta vritti nirodhah. Yoga* is a voluntary inhibition of irrelevant mental activity.) “We conceive of the right parieto-prefrontal network and its interactions with the midbrain arousal systems as the circuitry by which vigilant attention is maintained. This circuitry can be ‘tuned up’ (or down) endogenously or with exogenous support. Within this system, we believe that the prefrontal cortex plays a central role in maintaining and monitoring optimal arousal levels to match current task demands.” In their event-related functional MRI study, Garavan et al.[36] identified an activation of the right hemispheric network, during voluntary inhibition.

Mendez and Gershfield[37] described an overall neuroanatomical model of human attention. “Normal attention requires both the ascending reticular activating system (ARAS) in the upper brainstem and polymodal association areas of the cortex. Stimulation of the ARAS elicits arousal and primes the cortex for stimulus reception, whereas the polymodal association cortex controls and focuses this arousal energy for attending. These cortical areas are the ‘attentional gate’ for sensory input through feedback to the reticular nucleus of the thalamus… The important polymodal areas are the right prefrontal and the temporo-parietal cortex, although bi-hemispheric cooperation is needed for maintaining attention.” There are two phenomena at the limits of our attentional capacity: attentional blink when a second stimulus is missed if it is presented within 200 msec after the first stimulus and psychological refractory period when one is unable to respond to a second stimulus that occurs within 200 msec after the first response. There is also a phenomenon of central bottleneck as a limit to human information processing[38].

## SOCIAL-EMOTIONAL INTELLIGENCE

Emotional intelligence is defined as the competence to identify, understand, and express emotions; assimilate emotions in thought; and regulate both positive and negative emotions in oneself and others[39]. “Understanding the emotions of oneself and others is the key to a satisfying life. Those people who are self-aware and sensitive to others manage their affairs with wisdom and grace, even in adverse circumstances… Emotional intelligence is claimed to be positively related to academic achievement, occupational success and satisfaction and emotional health and adjustment.” Goleman[40] divided it into four domains: personal competence with self-awareness and self-management, and social competence with social awareness and relationship management. Bar-On[41] characterized it as an array of noncognitive capabilities, competencies, and skills that influence one's ability to succeed in coping with environmental demands and pressures. This is similar to the well-known yogic aphorism: *Yogah karmasu koushalam*, which means, *Yoga* is skill in action/behavior.

Panksepp[42] described the role of neocortex in emotion processing. "When the mushrooming of the cortex opened up the relatively closed circuits of our old mammalian and reptilian brains, we started to entertain alternatives of our own rather than nature's making. We can choose to enjoy fear. We can choose to make art of our loneliness. We can even exert some degree of control over our sexual orientations. Most other animals have no such options. Affectively, we can choose to be angels or devils, and we can construct or deconstruct ideas at will. We can choose to present ourselves in ways that are different from the ways we truly feel." Pritchard and Alloway[43] described the neural regulation of emotional behavior. "A rich supply of afferent projections from the association cortex provides the amygdaloid complex with visual, somato-sensory, auditory and viscero-sensory information. In addition, the amygdaloid complex has dense, reciprocal connections with hypothalamus and several brainstem nuclei, which allow it to coordinate a wide range of autonomic responses. These anatomic pathways suggest that amygdala plays a role in mediating emotion, personality, and other psychological processes."

Rauch et al.[44] observed increased brain blood flow in anterior cingulate, anterior temporal, and ventral globus pallidus regions, in eight healthy men during competitive and sexual arousal. Beauregard et al.[45] observed that right amygdala, right anterior temporal, and hypothalamus were activated during sexual arousal, whereas, right superior frontal and right anterior cingulate areas were activated during attempted inhibition of arousal. They conclude that humans have the capacity to influence the electrochemical dynamics of their brains, by voluntarily changing the nature of their mental processes. Baron-Cohen et al.[46] described a concept of "social brain", which mediates social intelligence. It consists of orbitofrontal cortex, superior temporal gyrus, and amygdala. Best et al.[47] provide evidence of dysfunction of the orbitomedial, prefrontal cortex in patients with impulsive aggressive behavior. Wild et al.[48] define the "laughter neural network". They summarize: "The expression of laughter seems to depend on two partially independent neural pathways. The first, an 'involuntary' or 'emotionally driven' system involves the amygdala, thalamic, hypo- and subthalamic areas and tegmental brainstem. The second, 'voluntary' (or a self-driven) system that originates in the premotor/frontal areas and leads through the motor cortex and pyramidal tract to the ventral brainstem. These systems appear to be coordinated by a laughter-coordinating center in the dorsal upper pons."

Heller et al.[49] have provided a synopsis of the relation between emotion and cognition and the skill of emotion regulation. "Human emotion can be conceptualized as a set of processes that include attention, appraisal, subjective feelings, and visceral and motor responses… The dorsolateral prefrontal cortex is implicated in executive control and working memory, whereas orbito-frontal cortex has been associated with emotional and affective processing… The right hemispheric system plays an executive role in modulating attention and vigilance in anxiety, especially in anxious arousal (i.e., panic and fear). This right hemisphere system promotes sympathetic nervous activity, spatial attention, visual scanning of the environment and sensitivity to meaningful nonverbal cues."

## THE SENSE OF SELF

The "sense of self" implies an intuitive perception and understanding of one's identity or selfness. According to the dictionary, the self is "the total, essential, or particular being of a person." Some of the recent publications in neuroscience on the subject of biophysical self are discussed below. Damasio[50] proposed three kinds of self, namely, proto-self, core-self and autobiographical self. "The proto-self is an interconnected and temporarily coherent collection of neural patterns, which represent the state of the organism, moment by moment, at multiple levels of the brain. We are not conscious of the proto-self… The core-self manifests as a second-order nonverbal account that occurs whenever an object modifies the proto-self. The core-self can be triggered by any object. The mechanism of production of core-self undergoes minimal changes across lifetime. We are conscious of the core-self… Because of the permanent availability of provoking objects, core-self is continuously generated and thus appears continuous in time. The mechanism of core-self requires the presence of proto-self. The autobiographical self is based on implicit memories of multiple instances of individual experience of the past and of the anticipated future. Autobiographical memory grows continuously with life experience… The autobiographical self requires the presence of core-self to begin its gradual development."

Llinas[51] argued that "prediction**,** which is a forecast of what is likely to occur, is the ultimate function of the brain. All moving animals use this predictive ability to efficiently guide their behavior in an ever-changing environment. Self is the centralization of prediction. Why this predictive ability arose is clear: it is critical to survival, guiding it at the level of both the single animal (moment-to-moment) and the species (in fact, of all actively moving species throughout evolution). 'I' has always been the magnificent mystery; I believe, I say, I whatever. But one must understand that there is no such tangible thing. It is just a particular mental state, a generated entity we refer to as 'I' or 'self'." Wegner[52], in his book *Illusion of Conscious Will,* stated: "Conscious will is something that is experienced when we perform an action – actions feel willed or not, and this feeling of voluntariness or doing a thing 'on purpose' is an indication of conscious will… Intention is normally understood as an idea of what one is going to do that appears in consciousness just before one does it."

Wegner and Sparrow[53] described "authorship processing supports the perception of events, actions and thoughts as issuing from the self as a causal agent. To discern whether a given item is authored by self or has arisen from some other source, this processing mechanism consults authorship indicators – sources of information about the likely origin of the item. The elemental indicators include body and direct bodily feedback, feed-forward, visual and other indirect sensory feedback, social cues, action consequences and action-relevant thoughts and environment orientation."

Kircher and David[54] proposed a model of consciousness and self-consciousness. "The contents of phenomenal consciousness are pre-reflexive, raw feelings qualia. They may be sensory experiences, memories and emotions. Their content is on a continuum of high or low self-valence. They may be conscious, when they are attended to; preconscious, when they are not attended to; or unconscious. Ipseity is the unifying 'basic tone' of the first-person giveness of all experiences. There is a special type of self-qualia, responsible for the feeling of unity, coherence, self-affectability and agency. If we reflect on qualia (i.e., think about primary experiences), the content enters introspective consciousness." Deshmukh[32] proposed the STEP (Situational, Transient, and Executive Process) model of self-consciousness. "Self-consciousness is transient and it arises from conscious attention and decision-making as a result of the sequential, executive activity in the frontal-parietal network in a given situation."

In describing the neuroanatomy of the self, Miller et al.[55] suggested that selfness depends on three core cognitive domains: (a) abstract/general information about personal attributes (semantic knowledge); (b) concrete, often affectively laden, experiences, on which self was formed (autobiographical memories); and (c) motivation to maintain self-schemas/images (will). They studied 72 patients with frontotemporal dementia who had impaired sense of self and found correlated changes in the right frontal region. Johnson et al.[56] describe the neural correlates of self-reflection. "The capacity to consciously reflect on one's sense of self is an important aspect of self-awareness. A sense of self is a collection of schemata regarding one's abilities, traits and attitudes that guide our behaviors, choices and social interactions." They studied functional MRI in healthy volunteers, and found that the medial prefrontal cortex and posterior cingulate areas were involved in self-reflecting process. Churchland[57] argued that "the self turns out to be identifiable not with a nonphysical soul, but rather with a set of self-representational capacities of the physical brain. Such capacities arose as evolution found neural network solutions for coordinating and regulating inner-body signals, thereby improving behavioral strategies."

LeDoux[58] suggested a synaptic view of the self. "Genes and experience or nature and nurture are, in the end, not different things, but different ways of doing the same thing – wiring the synapses of our brain… Obviously, much of who we are is based on memories learned through personal experience, including either conscious or unconscious memories." Zhu[59], in her essay "Locating Volition in the Brain", argued "volitions as acts of the will are special mental events or activities by which an agent consciously and actively exercises her/his agency to voluntarily direct her/his thoughts and actions…

Volition has at least three different components: 1) decision-making; 2) action initiation; and 3) executive control…The function of volition is supported by certain areas of the brain, particularly the anterior cingulate, the supplementary motor area and parts of the prefrontal cortex."

Ramachandran[24] explained: "Obviously self and qualia are two sides of the same coin. You can't have free-floating sensations or qualia with no one to experience them and you can't have a self completely devoid of sensory experiences, memories or emotions. The defining characteristics of the 'self' are fivefold. First of all continuity: a sense of unbroken thread running through the whole fabric of our experience with the accompanying feeling of past, present and future. Second is the idea of unity or coherence of self. Third is the sense of embodiment or ownership. Fourth is a sense of agency, what we call free will, being in charge of our own actions and destinies. Fifth, and most elusive of all, the self, almost by its very nature, is capable of reflection – of being aware of itself." Samson et al.[60] provide evidence that the inhibition of one's own point of view and the ability to infer someone else's point of view, rely on distinct neural processes. The right inferior frontal lobe is necessary for self-perspective inhibition, whereas, the left temporoparietal junction is necessary for inferring someone else's perspective. Turk et al.[61] present their evidence for the left hemisphere **"**interpreter**"** module, which generates a unitary sense of consciousness even in the disconnected brain and gives rise to a unified sense of self. According to Cavanna and Trimble[62], Precuneus plays a central role in visuospatial memory, episodic memory retrieval, and self-processing. It is a part of a neural network that mediates self-consciousness, engaged in self-related mental representations.

## THE MEDITATIVE MIND

From ancient times, people have been fascinated by the wonder of their own mind and the experience of the surrounding universe. This pondering, about one's being in reality, may have been the beginning of a meditative mind, and the origin of various philosophies and religions. Currently, there is a considerable interest among the medical and scientific community, in promoting mental health, subjective well-being, happiness, and spirituality. Vaillant[63] reviewed different models of mental health. He considered mental health as (a) normality, (b) positive psychology, (c) maturity, (d) social-emotional intelligence, (e) subjective well-being, and (f) resilience. Mental normality implies healthy ability to love, work, and play; efficient problem solving; investment in life; and self-autonomy. Klamut[64] defined happiness as a state of the spirit (mind), which consists of internal peace, satisfaction with one's life, the joy of life, benevolence, and cordiality towards oneself and others; sensitivity to the beauty of nature, culture, and art; harmonious coexistence with the surroundings. Achievement of such happiness depends on oneself and a rich, encouraging environment. McGrath[65] recently defined the term "spirituality" as a personal worldview (and self-view) that emphasizes the importance of discerning meaning in one's life.

The origin of yoga is difficult to establish since it was preserved mainly by oral tradition. There are references to *Yoga* in old *Sanskrit* literature like the *Svetashvatara* and *Maitri Upanishads,* and the *Bhagavad-Gita*. Ghosh[66], in his book *The Original Yoga*, included three books — *Shiva-Samhita*, *Gheranda-Samhita*, and *Patanjala Yogasutra* — as the basis of *Yoga*. Here are a few excerpts from the *Svetashvatara Upanishad*[67]: "let the wise man restrain his mind vigilantly as (he would) a chariot with vicious horses. (Verse 9). Even as a mirror stained by dust shines brightly when it has been cleaned, so the embodied one when he has seen the real nature of the Self becomes integrated, of fulfilled purpose and freed from sorrow. (Verse 14)." A few excerpts from *Maitri Upanishad*: "This is the rule for achieving this (oneness): control of breath, withdrawal of senses, meditation, concentration, contemplative inquiry, and absorption; (this is) said to be the six-fold yoga. (Verse 18)…That which is non-thought, which stands in the midst of thought, the unthinkable, the hidden, the highest, let a man merge his thought there. Then will this living being be (free) without any support (or dependence). (Verse 19)… For by the serenity of thought, one destroys deeds, good and evil, with the serene self abiding in itself, he enjoys eternal happiness. (Verse 20)."

A few excerpts from the sixth chapter of the *Bhagavad-Gita* on *Dhyana-Yoga*[68,69]: "Let a man lift his self by his own self; Let him not lower himself; One's self alone is one's own friend or foe. (Verse 5)… Unflickering, like a lamp in a sheltered place: So the man of disciplined thought practices yoga of the self. (Verse 19)… That in which thought ceases, stopped by the practice of disciplined concentration, and in which, seeing himself through himself, one is content in oneself; (Verse 20) The one who's self is disciplined by yoga, sees the self abiding in every being, and sees every being in the self. (Verse 29)." A few excerpts from *Shiva-Samhita*: "Out of consciousness is born all the animate and inanimate objects (experiences) of this world. Hence, abandoning all these, one should abide in consciousness itself. (Verse 49)… Indivisible by time and space, *Atman* in its natural state is always truly complete/whole. (Verse 54)… In a world full of ignorance and with extreme paucity of knowledge, happiness comes only when sorrow is removed. The *Atman*, however, is ever happy. (Verse 57)… The Atman alone is without duality and beyond words. (Verse 61)… All desires disappear only through knowledge (self-awareness). When the sense of plurality fades away, the light of Oneness dawns. (Verse 57)." *Gheranda-Samhita* describes a sixfold system of *yoga*: *Asanas* or postures, Mudras or mystic exercises, control of senses, breath control, meditation, and superconsciousness. Here are some excerpts: "Getting into (an absorptive) mental trance, let the mind be united with the self (*Atman*). When both (mind and self) merge into the supreme soul (*Paramatman*), *samadhi* ensues. (Verse 16)… All living creatures on land and in the sky, all trees, bushes, creepers, plants, grass, all rivers, and mountains – all are seen as the one supreme *Brahman*, and one's own self is seen in all. (Verse 19)."

*Yoga* is a way of adaptive self-integration, being at peace with oneself and at home in the world. Self-integration results from constant self-awareness and letting go of irrelevant memories, imaginations, mental agitations, and distractions. Such a person functions with a truly undistracted and serene mind. When the mind is undirected and assumes its original, unmodified state, the self experiences itself[70]. *Yoga* is a skillful quietening of a distracted and ruminative mind with a natural resolution of emotional conflicts, culminating in a truly peaceful and intuitive conscious state. Three essential steps of *Yoga* include (a) *Dharana:* conscious focusing of attention on an object, an activity, a thought or an ongoing experience; (b) *Dhyana***:** continued, sustained dwelling of attention on the same experience; and (c) *Samadhi:* effortless, self-absorptive, and intuitive state of understanding and realization of one's true nature or being. Here are a few excerpts from *Patanjali's Yoga-Sutra*: "*Yoga* is calming the fluctuations of a (distracted and agitated) mind (Verse 2). Then the self abides in his/her own (naturally serene) state (Verse 3). Other times, the self is associated with the agency (and ownership) of (ongoing) mental states (Verse 4)…Mental calming can be achieved by disengagement or letting go (of a ruminating mind) and constant self-awareness (presence, mindfulness or vigilant attentiveness) (Verse 12)… Impending pain and suffering can be prevented (by meditation) (Verse 67)… Self-regulatory activity and its continued experience as a controlling self are falsely associated. (Verse 74). The cause of selfishness or egotism is a lack of self-awareness (Verse 75). On becoming fully aware, the false association between self-regulatory activity and the essential self disappears, which leads to complete self-freedom (Verse 76). The uninterrupted deployment of vigilant awareness on the reality (presence or present-mindedness, vigilant attention) is the means of dispersing self-centered thinking and self-ignorance (Verse 77). With vigilant attentiveness comes fitness of mind (Verse 104)… The capacity to change mental state manifests in three different ways: ability to stop ongoing mental activity, ability to focus attention on a specific event in space-time, and the ability to maintain attention on a given task or event leading to enhanced cognitive capacity (Verse 122). Being free from any selfish motivation, instinctual pressure and mental conflict, one can live naturally with spontaneity, presence, and natural wisdom (Verse 195)."

There is a vast literature on Buddhist and Zen meditation. James Austin's book[71] *Zen and the Brain* is a landmark publication, integrating two very complex fields of knowledge. There are many insights in this book with attempts to understand meditation and consciousness in terms of neuroscience. However, it is beyond the scope of this article to review several, interesting observations made in that book.

Jiddu Krishnamurti (1895–1986) was a modern sage with a profound insight into the human mind. He was a great teacher who traveled tirelessly all over the world and shared his original insights. Here are a few of his excerpts[72]. "So there is an origin, an original ground, from which all things arise, and that original ground is not the word. The word is never the thing. And meditation is to come upon that ground which is the origin of all things and which is free from all time. This is the way of meditation. And blessed is he who finds it… Meditation means awareness – to be aware of what you are doing, what you are thinking, what you are feeling, aware without any choice, to observe, to learn… Out of this awareness comes the capacity to be completely attentive. Then there is freedom to see things as they actually are, without distortion (*tathata* or suchness in Buddhism). The mind becomes unconfused, clear, and sensitive; such meditation brings about a quality of the mind that is completely silent… There is no actual division between the organism and the mind. The brain, the nervous system, and the thing we call the mind are all one, indivisible. It is the natural act of meditation that brings about the harmonious movement of the whole… Meditation really is a complete emptying of the mind. Then there is only the functioning of the body; there is only the activity of the organism and nothing else; then thought functions without identification as the 'me' and the 'not me…' There is no self to understand but only the thought that creates the self… Mind can understand the present only if it does not compare, judge; the desire to alter or condemn the present without understanding it gives continuance to the past. Only in comprehending the reflection of the past in the mirror of the present, without distortion, is there renewal… We consider the present as a means to an end, so the present loses its immense significance. The present is the eternal."

## THE MEDITATIVE BRAIN

We do not know exactly what happens in the human brain during meditation. Obviously, it is difficult to bring together individuals accomplished in meditation, during their enlightened state of mind, in a high-tech laboratory with functional MRI, EEG, MEG, and PET scanning. Lazar et al.[73] used functional MRI to identify the brain regions that are active during a simple form of meditation and relaxation response. A significant increase in signals was observed in the dorsolateral prefrontal and parietal cortices, hippocampus, temporal lobe, anterior cingulate, striatum, and pre- and postcentral gyri during meditation. The results indicate that the practice of meditation activates neural structures involved in attention and control of the autonomic nervous system. Newberg and Iversen[74] described two types of meditation: "The first type is one in which the subjects simply attempt to clear all thought from their sphere of attention and to reach a subjective state, characterized by a sense of no space, no time, and no thought. This state is also fully integrated and unified, such that there is no sense of a self and other. The second form of meditation involves focusing attention on a particular object, image, word, or phrase, which may lead to a subjective experience of absorption with the object of focus. In eight Tibetan Buddhist monks, voluntary meditation with sustained attention was initially accompanied by activation in bilateral, but right more than left, prefrontal cortex and cingulate gyrus. This was followed by activation of the inhibitory reticular nucleus of the thalamus, which in turn deaffarented the posterior temporo-parieto-occipital areas, minimizing the (cortical) sensory input. The perception of one's bodily self depends on the activation of posterior, superior parietal lobules. The hippocampus acts to modulate cortical arousal and responsiveness via connections with the prefrontal cortex, amygdala, and hypothalamus. These structures are involved in generating attention, emotion, and imagery."

"Activation of right amygdala results in stimulation of the hypothalamus, with subsequent stimulation of the parasympathetic system. This is associated with a sense of relaxation and profound quiescence, reducing breathing and heart rates, which in turn, reduces the activity of locus ceruleus. Decreased NE from locus ceruleus would decrease the stimulation of hypothalamus, thus, decreasing the stress-related production of CRH, ACTH, and cortisol... Argenine vasopressin (AVP) is known to be released during meditation and has been shown to contribute to the maintenance of positive affect, to decrease self-perceived fatigue and arousal, and to significantly improve the consolidation of new memories and learning. The activation of prefrontal cortex increases the levels of free glutamate in the brain, which can stimulate the hypothalamus to release beta-endorphin. Beta-endorphin is known to reduce pain and fear, and to produce a sense of joy and euphoria. Moderately increased levels of serotonin, during meditation, also appear to correlate with positive affect."

McCraty et al.[75] define intuition as a process by which information that is normally outside the range of conscious awareness is perceived by the psychophysiological systems. They conclude that the heart and brain, together, are involved in receiving, processing, and decoding intuitive information. The intuitive perception and human creativity are closely linked[76]. Creativity is defined as the ability to produce work that is both novel (i.e., original, unexpected) and appropriate (i.e., useful, adaptive). Dietrich[77] summarized by stating, “Creativity results from the factorial combination of four kinds of mechanisms. Neural computation that generates novelty can occur during two modes of thought (deliberate and spontaneous) and for two types of information (emotional and cognitive). Regardless of how novelty is generated initially, circuits in the prefrontal cortex perform the computation that transforms the novelty into creative behavior. To that end, prefrontal circuits are involved in making novelty fully conscious, evaluating its appropriateness, and ultimately implementing its creative expression.”

## NEW HYPOTHESIS

Meditation is an art of efficient and adaptive management of neurobehavioral energy with total engagement or disengagement. In both of these conscious states, there is an experience of spontaneous, unitive being without a sense of ego, memory, or time. There is a natural sense of well-being with self-understanding, spontaneous joy, serenity, freedom, and self-fulfillment. It is where the ultimate pursuit of happiness and search for meaning in life resolve. One realizes the truth of one’s being in harmony with nature and nature in oneself. Then each conscious moment becomes a dynamic process of discovery and learning of the ever-new reality. One’s daily living becomes joyful (*Ananda*) and ever enlightening beyond all measure and description.

Attention can be voluntarily or involuntarily engaged or disengaged. An attentional act creates the duality of the attended-attendee, resulting in a specific conscious experience. Disengaging attention to the limit results in a resting attentiveness with global, nonspecific (nirguna) meta-awareness (awareness of awareness). It may be expressed as the great nothingness (*shunyata*), silence, or the essential consciousness[70]. Engaging attention to the limit, with a total participation in the present moment, without any effort, sense of time, or self-consciousness, results in a conscious totality (*poornata*), the “flow” state[78] or “presence”[70]. Between two consecutive attentional acts, there is an inter-attentional awareness, with no attended-attendee duality. This results in a refreshing experience of no experience, which is analogous to waking up from a sound, non-REM sleep with no dreams. This inter-attentional awareness can lead to a state of non-REM alertness or *Turiya*. During non-REM alertness and non-REM sleep, conscious mentation exists only in a potential form.

Neurobehavioral energy and mentation can be in a potential or kinetic form. They are in kinetic form during common, interactive consciousness and dream sleep, whereas, during non-REM sleep and non-REM alertness (*turiya*), they are in a potential form. During non-REM sleep, the conscious arousal is low, whereas during non-REM alertness, the conscious arousal is high. That is why the *turiya* state has been described to have a sense of boundless energy, unlimited capacity, and an intense sense of well-being. This enlightening process is nonlinear and dynamic, whereas the common state of self-consciousness functions within a chaotic attractor basin[32,79,80]. The moment one resumes directed attention, the subject-object duality returns and the “potential” neurobehavioral energy becomes “kinetic” and the egoistic mind takes on various conscious experiences. The ego continuously struggles to change the situation or itself, and suffers from pain and pleasure. Meditation, on the other hand, is the art of being alert and serene in the midst of any situation. It is the ultimate, adaptive reality-consciousness, continuously learning and renewing itself. It is the art of mastering both skills of simultaneous as well as sequential conscious processing from moment to moment. It is the most optimal state of one’s being at each moment, discovering and appreciating the wonders of this infinite, ever-new reality.

## ACKNOWLEDGMENTS

My special thanks to Sunanda V. Deshmukh, MD for a careful review and good suggestions for the article.
